# The Influence of Lithium and/or Selenium Treatment on Homeostasis of Chosen Bioelements in Rats

**DOI:** 10.1007/s12011-016-0906-x

**Published:** 2016-12-12

**Authors:** Małgorzata Kiełczykowska, Irena Musik, Jacek Kurzepa, Renata Żelazowska, Anna Lewandowska, Marek Paździor, Joanna Kocot

**Affiliations:** 1 Chair and Department of Medical Chemistry, Medical University of Lublin, Chodźki 4a, 20-093 Lublin, Poland; 2 Traumatic-Orthopaedic and Spine Surgery Ward of Independent Public Health Care Centre in Puławy, Józefa Bema 1, 24-100 Puławy, Poland

**Keywords:** Lithium, Selenium, Magnesium, Calcium, Silicon, Male rats

## Abstract

Lithium is widely used in medicine and the therapy is often long term. Apart from beneficial effects, its application can cause diverse side effects. The current study was performed with the aim of the evaluation of the effect of lithium and/or selenium administration on magnesium, calcium and silicon levels in rats. The study was performed on rats divided into four groups (six animals each): control—received saline, Li—received Li_2_CO_3_ (2.7 mg Li/kg b.w.), Se—received Na_2_SeO_3_·H_2_O (0.5 mg Se/kg b.w.), and Li+Se—received simultaneously Li_2_CO_3_ and Na_2_SeO_3_·H_2_O (2.7 and 0.5 mg Se/kg b.w.). The administration was performed in form of water solutions by a stomach tube once a day for 6 weeks. In the organs (liver, kidney, brain, spleen, heart, lung and femoral muscle), the concentrations of magnesium, calcium and silicon were determined. Lithium significantly increased Ca in the kidney, brain and spleen. Coadministration of selenium reversed this effect. No changes of magnesium in organs were observed. Silicon was affected only in spleen—an increase vs. control was observed in all studied groups. The beneficial influence of coadministration of selenium in case of calcium lets us suggest that an issue of its possible use as an adjuvant alleviating side effects in lithium-treated subjects is worth being continued.

## Introduction

For many years, lithium carbonate has been used in medicine, mostly in psychiatry [1, 2]. However, studies have revealed the possibility of its application in other fields, e.g. in cure of thyroid and neurodegenerative disturbances [2–4]. Lithium chloride application in turn has been suggested to be effective in leukaemia therapy [5]. Apart from a beneficial influence, lithium therapy can be accompanied with diverse side effects including parathyroid, renal and thyroid disorders [6, 7]. The additional impediment for the clinicians is the fact that lithium is characterized by a narrow therapeutic range which must not be exceeded [8]. However, in cases of bipolar disorder, lithium therapy remains a first choice maintenance treatment [9].

The research on an adjuvant which could alleviate lithium’s side effects seems to be worth performing, all the more that the therapy is often long term. Moreover, the possibility of growing environmental pollution with lithium, resulting from its growing use, has been reported [10]. Harrari et al. have reported that the lithium occurrence in drinking water should also be considered as a potential threat for consumers [11]. Having regarded that diverse bioelements proved to protect against different harmful factors, including chemical substances of toxic properties, we chose selenium to be studied in regard to its possible beneficial influence on organisms subjected to lithium. Selenium has already been found to show protective action against both physical and chemical factors, as well as against side effects of drugs [12–14]. Additionally, an organic compound ebselen containing selenium has been proved to possess some lithium-mimetic properties [15]. As to the used form of selenium, for this pilotage study, an inorganic sodium selenite has been chosen, considering its bioavailability and continuous application in animal studies as well as in clinical practice [16, 17].

Lithium has already been reported to show relationships with homeostasis of different bioelements, both macro- and microelements [11, 18, 19]. Lithium and magnesium ions have been reported to compete for binding site of some enzymes [20]. Neuroprotective action of lithium has been suggested to be linked with the influence on intracellular concentrations of calcium ions [3]. Hypercalcaemia may occur in lithium-received subjects and despite investigations, the mechanism of this effect has not been fully clarified yet [6, 21]. Silicon, an essential microelement has been found to be influenced by lithium administration [19].

The current study was performed with the aim of evaluating the effect of lithium administration on chosen bioelements—magnesium, calcium and silicon—in rats as well as the possibility of selenium application as an adjuvant in lithium therapy.

## Materials and Methods

### Experimental Animals

The experiment was carried on adolescent male Wistar rats (24 animals, 130–160 g body weight). The animals had free access to standard feed (LSM produced by AGROPOL S.J., Motycz, Poland, without lithium and selenium supplementation) and drinking water. The composition of the diet is presented in Table 1.

**Table 1:** Composition of standard LSM feed

Substance	Unit	Content
Protein min.	[%]	16.00
Raw fats min.	[%]	2.80
Raw ash max.	[%]	7.00
Raw fibre max.	[%]	5.00
L-Lysine min.	[%]	0.80
DL-Methionine min.	[%]	0.50
Calcium min.	[%]	1.10
Phosphorus min.	[%]	0.60
Sodium max.	[%]	0.20
Vitamin A (retinol) E 672	[jm/kg]	10,000
Vitamin D3 E 671	[jm/kg]	1500
Vitamin E (α-tocopherol 50%)	[mg/kg]	25
Copper (copper sulphate 24.5%) E 4	[mg/kg]	5

The study was performed according to statutory bioethical standards and approved by I Local Ethical Commission of Medical University of Lublin, acceptance no.1/2013.

### Experimental Design

After an acclimatization period of 3 days, the animals were divided randomly into four groups (six animals each). The treatments were as follows:

- control group—treated with saline,
- Li group—treated with lithium (as Li_2_CO_3_) at a dose of 2.7 mg Li/kg b.w.,
- Se group—treated with selenium (as Na_2_SeO_3_·H_2_O) at a dose of 0.5 mg Se/kg b.w.,
- Li+Se group—treated simultaneously with lithium (Li_2_CO_3_) and selenium (Na_2_SeO_3_·H_2_O) at a dose of 2.7 mg Li/kg b.w. and of 0.5 mg Se/kg b.w., respectively.

The administration was performed in form of water solutions by a stomach tube, once a day, for a period of 6 weeks. Basing on the body mass of each animal, measured every day before administration, the appropriate amount of selenium and/or lithium solutions was calculated. Then the animals were sacrificed under thiopental narcosis and samples of the liver, kidney, brain, spleen, heart, femoral muscle and lung were collected. The organs were weighed when collected. Ten per cent (*w*/*v*) tissue homogenates were prepared in 0.1 mol dm^−3^ Tris-HCl buffer, pH = 7.4. Supernatants were obtained by centrifugation at 5000×*g* for 30 min.

### Tissue Bioelement Analysis

In the prepared tissue supernatants, the concentrations of magnesium, calcium and silicon were determined. Magnesium and calcium concentrations were assayed by colorimetric methods using diagnostic kits Liquick Cor-MG 60 and Liquick Cor-CALCIUM 120, respectively.

The concentration of silicon was measured using the spectrophotometric method described by Wielkoszyński [22]. Tissue homogenates were mixed with 10% trichloroacetic acid (TCA) solution in a volume ratio 1:1.6 and left for 30 min with the aim of deproteination. Then the samples were centrifuged (13,000×*g*, 10 min) and 0.5 cm^3^ of supernatant was collected. Then 1 cm^3^ of 0.5% solution of ammonium molybdate (NH_4_)_6_Mo_7_O_24_·4H_2_O in 30% acetic acid was added and the samples were kept in water bath (80 °C) for 5 min. After cooling, 0.5 cm^3^ of 4% sodium potassium tartrate solution in 4 M H_2_SO_4_ and, after subsequent 2 min, 1 cm^3^ of 2% water solution of ascorbic acid were added. After the next 5 min, the absorption of the obtained samples was read at wavelength 800 nm against the blank prepared as described above with using distilled water instead of tissue homogenates. Silicon concentration was measured basing on the standard curve prepared with using silicon standard Titrisol-Silicium (Merck).

The obtained values of the determined elements’ concentrations were expressed in micromoles per gram of wet tissue. The assays were performed using spectrophotometer SPECORD M40 (Zeiss Jena).

### Statistical Analysis

All statistical analyses were performed using the STATISTICA program (version 10.0). The normality of data distribution was verified using the Shapiro-Wilk test. The differences among the studied groups were analysed using a one-way analysis of variance (ANOVA), followed by the Tukey test (for normally distributed variables) or Kruskal-Wallis one-way analysis of variance (for non-normally distributed variables). Values were considered significant with *p* < 0.05.

## Results

### Body Weight Gain, Feed and Water Intake and Organ Weights of the Experimental Animals

**Fig. 1:**
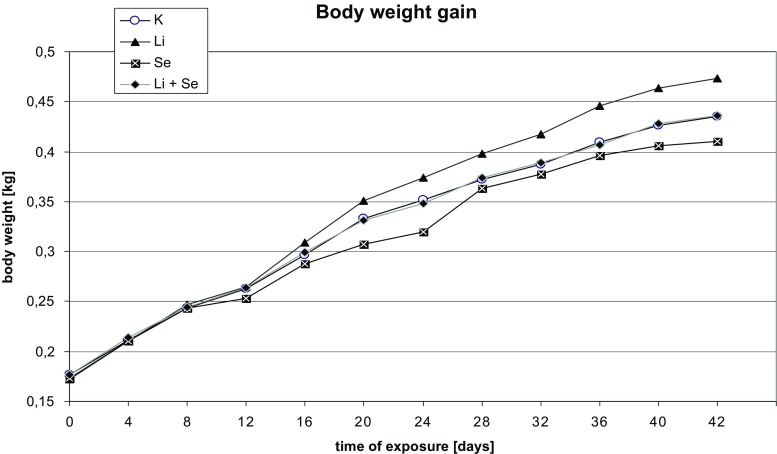
Body weight gain of experimental animals

The experimental animals’ body weight enhanced during all experiments showing no statistical differences among the groups. However, the slight increase in Li-treated rats as well as a little depletion in Se-given rats vs. control was observed (see Fig. 1).

The values of intake of feed and water showed no differences among the control and the studied groups of rats (Li treated, Se treated and Li+Se treated) during all the experiments.

Generally, the weight of the studied organs did not show significant differences among the studied groups, except for liver and kidney, where a significant decrease in Li-treated animals vs. control was observed. Additionally, liver weights in Se-alone and Li+Se-treated groups were significantly enhanced compared to the Li-alone group (see Table 2).

**Table 2:** Organ weight [g] of rats receiving lithium and/or selenium

Group	Liver	Kidney	Brain	Spleen	Heart	Lung
C (*n* = 6)	7.9 ± 0.7	2.1 ± 0.2	1.3 ± 0.3	0.6 ± 0.2	0.7 ± 0.2	1.7 ± 0.1
Li (*n* = 6)	5.5 ± 0.4**	1.4 ± 0.4*	1.0 ± 0.3	0.8 ± 0.3	0.6 ± 0.3	1.6 ± 0.1
Se (*n* = 6)	7.4 ± 0.8^##^	2.0 ± 0.3	1.2 ± 0.8	0.6 ± 0.2	0.6 ± 0.3	1.8 ± 0.2
Li+Se (*n* = 6)	7.3 ± 0.6^##^	1.9 ± 0.3	1.2 ± 0.7	0.6 ± 0.2	0.7 ± 0.4	1.7 ± 0.3

Values are mean ± SD

*p < 0.05 vs. control; **p < 0.01 vs. control; ^**##**^ *p* < 0.01 vs. Li group

### Calcium Level in Organs of Rats

Calcium level in the studied organs was considerably influenced. In the liver, it was decreased slightly by Li alone and significantly by Se alone compared to control. Coadministration of these both elements did not change liver Ca vs. control but a significant increase vs. Se alone was observed. In the kidney, lithium markedly increased Ca vs. control. Se, both alone and given with Li, did not alter Ca vs. control but caused a significant decrease compared to the Li group. In the brain, Li alone resulted in a well-marked enhancement of Ca vs. control. The similar significant Ca enhancement vs. control was observed in the spleen, but in this organ, Se and Li+Se treatment caused a well-marked depletion compared to the Li group. In the heart, Li alone caused an insignificant Ca decrease vs. control. Se, both alone and given with Li, significantly increased Ca compared to Li group. In the lung, a slight Li increase vs. control was observed, whereas in the Li+Se group, a depletion vs. the Li group was noted. In femoral muscle, selenium significantly increased Ca vs. all the other groups. All the presented results are shown below (see Table 3).

**Table 3:** Calcium concentrations [μmol g^−1^ of wet tissue] in organs of rats receiving lithium and/or selenium

Group	Liver	Kidney	Brain	Spleen	Heart	Lung	Femoral muscle
Control (*n* = 6)	3.6 ± 0.6	6.9 ± 1.3	3.0 ± 0.7	7.8 ± 1.6	8.1 ± 2.9	9.0 ± 2.5	2.6 ± 0.6
Li (*n* = 6)	2.8 ± 0.8	10.6 ± 1.1***	5.3 ± 2.4*	12.1 ± 1.7***	5.2 ± 1.9	10.7 ± 1.4	3.9 ± 1.0
Se (*n* = 6)	1.8 ± 0.5*	6.4 ± 1.2^**###**^	3.0 ± 0.6	7.9 ± 0.4^**###**^	10.9 ± 1.8^**##**^	8.0 ± 1.8	7.7 ± 1.6***^, **###**^
Li+Se (*n* = 6)	4.2 ± 1.8^####^	5.1 ± 1.0^**###**^	4.9 ± 0.6	8.0 ± 0.8^**###**^	10.0 ± 1.9^**##**^	7.6 ± 1.7^#^	3.7 ± 0.6^#####^

Values are mean ± SD

*p < 0.05 vs. control; ***p < 0.001 vs. control; ^**#**^ *p* < 0.05 vs. Li group; ^**##**^ *p* < 0.01 vs. Li group; ^**###**^ *p* < 0.001 vs. Li group; ^####^ *p* < 0.05 vs. Se group; ^#####^ *p* < 0.001 vs. Se group

### Magnesium Level in Organs of Rats

In all the studied organs, magnesium was not disturbed significantly by any used treatment (see Table 4).

**Table 4:** Magnesium concentrations [μmol g^−1^ of wet tissue] in organs of rats receiving lithium and/or selenium

Group	Liver	Kidney	Brain	Spleen	Heart	Lung	Femoral muscle
C (*n* = 6)	7.6 ± 1.0	7.0 ± 1.1	3.4 ± 0.4	8.3 ± 0.4	7.9 ± 0.6	4.8 ± 0.5	11.1 ± 0.7
Li (*n* = 6)	7.6 ± 0.5	7.8 ± 0.7	3.6 ± 0.7	8.6 ± 0.7	8.0 ± 0.6	5.0 ± 0.3	11.2 ± 2.4
Se (*n* = 6)	7.6 ± 0.9	6.9 ± 0.5	3.6 ± 0.2	7.5 ± 0.6	8.2 ± 0.3	4.7 ± 1.6	12.1 ± 0.7
Li+Se (*n* = 6)	8.2 ± 0.6	7.4 ± 0.5	3.5 ± 0.4	8.0 ± 0.2	8.2 ± 1.0	4.4 ± 0.4	11.3 ± 0.7

Values are mean ± SD

### Silicon Level in Organs of Rats

Silicon level was markedly affected only in spleen where all the treatments significantly increased its level vs. control. Additionally, in the Se-alone group, Si was enhanced compared to the Li group. In the other organs, no significant differences among the studied groups were observed. The results are shown below (see Table 5).

**Table 5:** Silicon concentrations [μmol g^−1^ of wet tissue] in organs of rats receiving lithium and/or selenium

Group	Liver	Kidney	Brain	Spleen	Heart	Lung	Femoral muscle
C (*n* = 6)	3.4 ± 1.2	5.1 ± 0.8	0.9 ± 0.3	1.7 ± 0.3	0.5 ± 0.1	1.0 ± 0.2	1.6 ± 0.3
Li (*n* = 6)	2.8 ± 0.5	3.7 ± 0.9	0.7 ± 0.1	2.4 ± 0.4*	0.7 ± 0.3	1.1 ± 0.4	1.1 ± 0.4
Se (*n* = 6)	3.5 ± 0.8	5.1 ± 1.2	1.1 ± 0.5	3.2 ± 0.2***^, **#**^	0.5 ± 0.1	0.8 ± 0.4	1.2 ± 0.4
Li+Se (*n* = 6)	3.3 ± 0.8	4.5 ± 1.1	0.9 ± 0.3	2.5 ± 0.6*	0.6 ± 0.2	1.1 ± 0.3	1.3 ± 0.4

Values are mean ± SD

*p < 0.05 vs. control; ***p < 0.001 vs. control; ^**#**^ *p* < 0.05 vs. Li group

## Discussion

Lithium treatment applied in the current study, although rather short compared to therapy used in humans, resulted in a slight increase in body weight vs. control. It is consistent with the findings revealing the occurrence of body weight gain in patients undergoing lithium therapy [7].

In the previous part of our research, we found that lithium treatment significantly decreased serum Mg and enhanced serum Ca [23]. In contrast, in the current study, magnesium in organs was not affected by Li treatment, whereas calcium was changed in the studied organs in different ways. These results suggest that alterations observed in serum do not fully reflect changes of these elements’ homeostasis in all organisms. Such observations seem to be important and point to the usefulness of the animal model studies, as lithium therapy is applied in humans and the only possible monitoring of its effect is the determination of blood parameters. Moreover, the results concerning lithium’s influence on blood calcium are not consistent. Although hypercalcaemia is included into side effects of Li therapy, Zamani et al. did not observe any difference in corrected total calcium between patients receiving maintenance lithium therapy and healthy control [1]. Harrari et al. reported a depletion of urinary calcium across the tertiles of lithium concentration in the blood of pregnant women, inhabitants of an area with Li-containing drinking water. However, no changes of total serum calcium were observed. The authors suggested that the disturbances of calcium homeostasis were connected with vitamin D [11] what is consistent taking into account that kidney disorders are one of the main negative effects of lithium administration [24]. Other studies also revealed the occurrence of vitamin D disturbances in subjects treated with lithium [25].

In the current study, calcium was the most affected bioelement in organs by lithium and the prevailing effect was an increase vs. control. The relationships between the homeostasis of this element and lithium have already been reported, and the results were not consistent as well as showed time dependence. Baltaci et al. observed a significant increase in bone Li in ovariectomized rats fed zinc-deficient diet, and this effect was accompanied with a well-marked depletion of bone calcium [26]. Tkatcheva et al. found a time dependence of lithium’s influence on calcium in fish brain. While at the beginning, Ca remained unaltered; after 24 h, a slight decrease compared to control was obtained. The lengthening of intoxication up to 72 h resulted in a slight increase but after 96 h, no changes were observed again [10]. Another study revealed a dentin Ca decrease in a patient undergoing Li therapy [27].

Irrespective of the studied organ, in the present experiment, no used treatment affected the magnesium tissue level. The lack of brain Mg changes seems to be vital since magnesium is considered to be one of the most important elements for the central nervous system [28]. Our results are supported by the outcomes reported by other researchers. According to Sivrikaya, intraperitoneal sodium selenite did not influence Mg in rat liver [29]. One of our previous experiments revealed no effect or increase in tissue Mg in rats receiving the similar Li doses in drinking water [30]. LiCl provided with drinking water did not alter magnesium in chosen organs (brain, liver, skeletal muscle and cardiac muscle) of rats [31]. A study carried out on ovariectomized rats fed Zn-deficient diet showed that while bone Li was increased, no alterations of bone magnesium were observed [26]. However, other scientists reported the time-dependent lithium’s effect on brain magnesium in fish. During the first 48 h of the experiment, no alterations were observed, but after 72 and 96 h, a slight decrease vs. control was obtained [10]. No influence of any treatment used in our experiment on the liver Mg is also very substantial as the proper functioning of this detoxifying organ is an issue of great importance, particularly that psychiatric subjects must undergo a long-term therapy [2] and some connections among magnesium, selenium and disturbances of the liver have already been reported [32, 33].

The human studies revealed little relationships between Ca, Mg and selenium in organism. Özkaya et al. found that in women undergoing in vitro fertilization and receiving a multivitamin/mineral supplementation (containing 100 mg of Mg and 125 mg of Ca per 45 days), a considerable selenium increase in follicular fluid was accompanied with no effect on magnesium and calcium [34]. Crujeiras et al. found no changes of blood calcium in patients with hyperphenylalaninemia, although 25% of them showed depressed selenium [35]. In a human study concerning the relationships between these two elements, no effect of calcium supplementation on selenium parameters in adolescent girls was observed [36]. It can be another confirmation of usefulness of animal model.

The current study revealed that the administration of lithium and/or selenium did not affect silicon in any organ except for the spleen. The lack of any effect in the case of the brain seems to be worth underlying as lithium is used in psychiatric and neurological patients and silicon is considered to be essential for brain functions [37]. Generally, little information concerning relationships among these three bioelements can be found in the available literature data. In our department, the influence of lithium provided in drinking water on silicon in chosen rat organs was studied, revealing that comparable Li doses resulted mostly in no effect. A slightly higher dose enhanced Si in the liver and femoral muscle, whereas a lower one depleted the kidney Si level. But, first of all, a wide range of Li doses, applied in the mentioned study, did not change silicon in the brain [19]. As to the connections between selenium and silicon, this issue has been poorly investigated by to date. However, a silicon influx transporter OsNIP2;1 was suggested to be the first transporter of inorganic selenium (selenite form) in different organisms—plants, microorganisms and animals [38].

As side effects of lithium may considerably influence life condition of patients as well as their compliance, the research on agents alleviating lithium’s negative influence has already been performed. Zinc was shown to be effective in improving of the disturbances of oxidant parameters, resulted from lithium exposure, in erythrocytes [39] and partially in the liver [40]. Some substances of natural origin, possessing the antioxidant properties, showed the similar protective influence in the kidney [41] and liver [42].

In the current experiment, the lithium-induced changes of studied parameters were reversed by coadministration of selenium, except for silicon in the spleen. These results let us suggest that selenium could be taking into account as an adjunct to lithium therapy, and farther studies of this issue seem to be worth undertaking.
